# Exogenous Hydrogen Sulfide Plays an Important Role by Regulating Autophagy in Diabetic-Related Diseases

**DOI:** 10.3390/ijms22136715

**Published:** 2021-06-23

**Authors:** Shuangyu Lv, Huiyang Liu, Honggang Wang

**Affiliations:** Henan International Joint Laboratory of Nuclear Protein Regulation, School of Basic Medical Sciences, Henan University, Kaifeng 475000, China; shuangyulv@henu.edu.cn (S.L.); m15736875597@163.com (H.L.)

**Keywords:** hydrogen sulfide, autophagy, diabetes, signaling pathway, oxidative stress

## Abstract

Autophagy is a vital cell mechanism which plays an important role in many physiological processes including clearing long-lived, accumulated and misfolded proteins, removing damaged organelles and regulating growth and aging. Autophagy also participates in a variety of biological functions, such as development, cell differentiation, resistance to pathogens and nutritional hunger. Recently, autophagy has been reported to be involved in diabetes, but the mechanism is not fully understood. Hydrogen sulfide (H_2_S) is a colorless, water-soluble, flammable gas with the typical odor of rotten eggs, which has been known as a highly toxic gas for many years. However, it has been reported recently that H_2_S, together with nitric oxide and carbon monoxide, is an important gas signal transduction molecule. H_2_S has been reported to play a protective role in many diabetes-related diseases, but the mechanism is not fully clear. Recent studies indicate that H_2_S plays an important role by regulating autophagy in many diseases including cancer, tissue fibrosis diseases and glycometabolic diseases; however, the related mechanism has not been fully studied. In this review, we summarize recent research on the role of H_2_S in regulating autophagy in diabetic-related diseases to provide references for future related research.

## 1. Introduction

**Figure 1:**
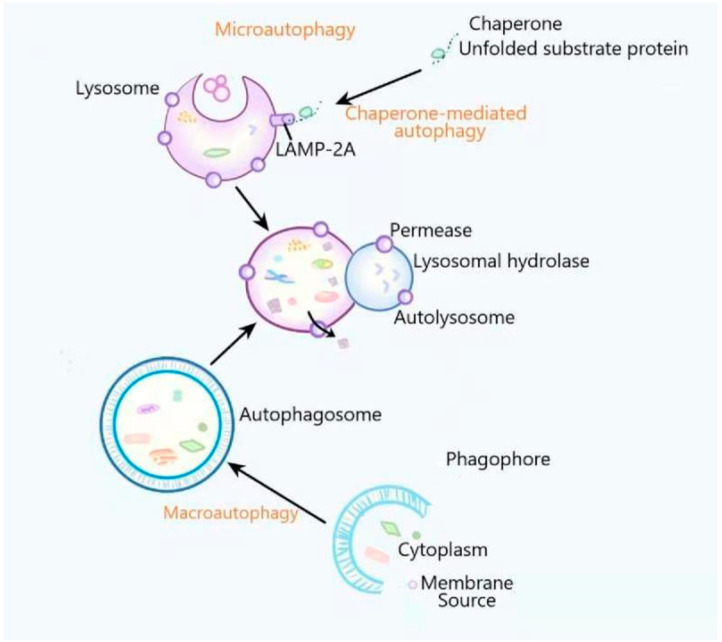
The general process of macroautophagy, microautophagy and chaperone-mediated autophagy. In the process of macroautophagy, the content is wrapped by a bilayer membrane structure to form an autophagosome and then fuses with lysosomes for degradation. Microautophagy refers to the process by which the lysosomal membranes directly invaginate and then encapsulate the cell contents. In the process of chaperone-mediated autophagy, the cytosolic proteins are transported to the lysosomal chamber after binding to molecular chaperones and then are digested by lysosomal enzymes.

Autophagy is a closely coordinated process that isolates proteins and damaged or aged organelles in double-membrane vesicles called autophagosomes, which eventually fuse with lysosomes, leading to the degradation of the isolated components [1]. According to the type of degraded cargo and the way of transporting cargo to lysosomes, autophagy can be divided into three types: macroautophagy, microautophagy and chaperone-mediated autophagy. Macroautophagy, which is the most common autophagy, promotes autophagosome formation. The autophagosome is a cytosolic double-membranous vesicle which isolates part of the cytoplasm. Autophagosomes then fuse with lysosomes to form autolysosomes in which the isolated cytoplasm is degraded or recycled [2,3]. Microautophagy refers to the direct invagination of a lysosomal membrane, which then encapsulates the cell contents [4]. Chaperone-mediated autophagy is a type of selective autophagy, in which proteins in cells are transported to lysosomal chambers after binding with chaperones and then digested by lysosomal enzymes [5,6] (Figure 1). Autophagy is the main cellular pathway regulating the degradation of long-lived proteins and the only known degradation pathway of cytoplasmic organelles. It has been reported that autophagy and the ubiquitin–proteasome system (UPS) are two important quality control systems for degrading proteins and organelles in eukaryotic cells [7]. Autophagy includes several successive steps: induction, autophagosome formation, autophagosome fusion and degradation [8]. Beclin1, LC3, P62 and other conserved proteins participate in the autophagy process and are regarded as autophagy-related proteins [9]. Among them, LC3, a ubiquitin-like protein, promotes the formation of autophagosomes [8,10]. It regulates the elongation and closure of the autophagic membrane by binding with phosphatidylethanolamine [11]. Autophagy is influenced by many factors including immune or inflammatory stimulation, endoplasmic reticulum stress, Ca^2+^ concentration, nutritional deficiency and accumulation of damaged cells or organelles [12,13]. Autophagy is usually maintained at the basic level under physiological conditions. In the pathological state, upregulated autophagy can clear the abnormal proteins in cells to help cell survival [14]. However, if autophagy remains at a high level, autophagy will induce cell death [15,16]. Autophagy plays a vital role in many physiological processes and diseases, including the immune response, starvation adaptation, development, quality control of intracellular proteins and organelles, anti-aging mechanisms, tumor suppression [13,17,18,19], cardiovascular disease [20], neurodegenerative diseases [21], infection and immunity [17]. However, the related mechanisms are not fully understood.

Diabetes is an important metabolic disease. Its prevalence rate is significantly higher than before. In the 1990s, the number of diabetic patients worldwide was 135 million, and it may rise to 300 million by 2025 [22]. Diabetes is classified as type 1 diabetes and type 2 diabetes. Insulin-dependent diabetes mellitus (also known as type 1 diabetes) is sensitive to insulin therapy. The incidence of the disease is closely related to genetic factors. Most patients belong to autoimmune diseases. Insulin antibodies are found in the serum of patients, which renders insulin unable to play its normal biological activities. The patient’s insulin secretion gradually decreases until it is completely lost and an insulin supplement is needed. Patients with typical clinical symptoms and a serious condition are prone to ketoacidosis, and even coma. Noninsulin-dependent diabetes mellitus (also known as type 2 diabetes) is commonly seen in obese adults. These patients’ blood insulin level is not low, but insulin receptor deficiency leads to a poor response of target cells to insulin. Type 2 diabetic patients have mild clinical symptoms and no ketoacidosis. They are not sensitive to insulin treatment [23,24].

**Figure 2:**
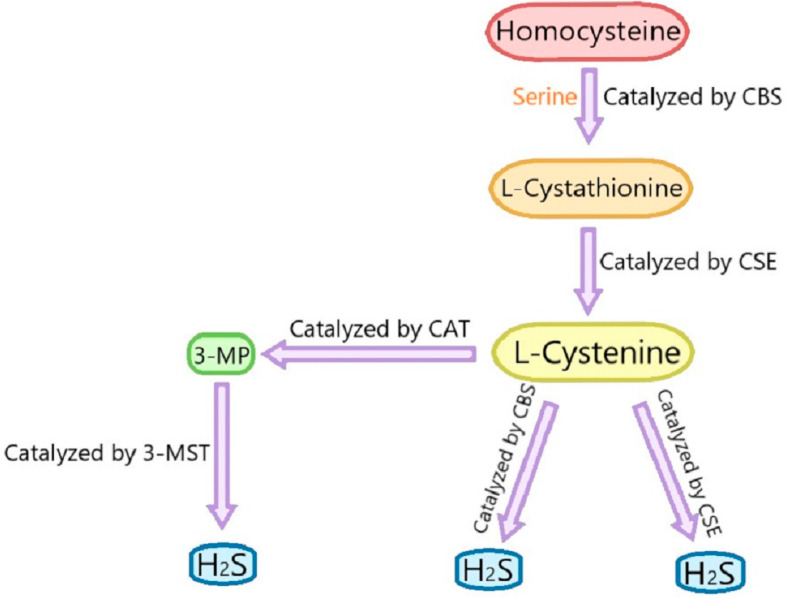
Summary of the production process of endogenous H_2_S. CBS: cystathionine-beta-synthase; CSE: cystathionine-gamma-lyase; 3-MST: 3-mercaptopyruvate thiotransferase; 3-MP: 3-mercaptopyruvate; CAT: cysteine aminotransferase.

For decades, hydrogen sulfide (H_2_S) has been understood as a colorless gas with a smell of rotten eggs, which is recognized as a toxic gas and environmental pollutant. Recently, along with carbon monoxide (CO) and nitric oxide (NO), H_2_S is considered as the third gasotransmitter [25]. Endogenous H_2_S is produced from L-cysteine and/or L-homocysteine catalyzed by cystathionine γ-lyase (CSE), cystathionine β-synthase (CBS), cysteine aminotransferase and 3-mercaptopyruvate sulfurtransferase (3-MST) [26]. Cystathionine is produced by the β-substitution reaction of homocysteine with serine catalyzed by CBS. CSE catalyzes the elimination of α, γ-cysteine of cystathionine to produce cystenine. Under the catalysis of CBS and CSE, cysteine can form H2S through the β-elimination reaction. 3-mercaptopyruvate (3-MP) is produced by transferring amines from cystine to α-ketoglutarate via cysteine aminotransferase (CAT). 3-MST catalyzes the sulfur of 3-MP to convert into H_2_S [27] (Figure 2). H_2_S has been reported to have many biological functions including antiapoptosis [28], antioxidative stress [29], relaxing blood vessels, lowering blood pressure [30,31] and anti-inflammation [32]. The excessive production of reactive oxygen species (ROS) leads to increased oxidative stress, which is involved in the occurrence of many chronic diseases [33]. Therefore, the antioxidant effect of H_2_S is particularly important.

It has been reported that exogenous H_2_S improves diabetes-accelerated atherosclerosis through inhibiting oxidative stress via Kelch-like ECH-associated protein 1(Keap1) sulfhydrylation of Cys151 to activate nuclear factor erythroid-2 related factor 2(Nrf2) signaling [34]. H_2_S also alleviates diabetes-induced atrial remodeling and atrial fibrillation by activating the phosphatidylinositol 3 kinase (PI3K)/protein kinase B (Akt)/eNOS pathway [35]. At present, the important mechanism of H_2_S affecting diabetes is the effect of H_2_S on the pancreas. H_2_S can be produced in the pancreas. In pancreatic beta cells, H_2_S inhibits high glucose (HG)-induced insulin release and decreases HG-induced apoptosis of pancreatic islets. H_2_S also protects pancreatic beta cells against glucotoxicity by increasing glutathione content and reducing ROS production. However, the high concentrations of H_2_S can promote the apoptosis of pancreatic beta cells [36]. Endogenous H_2_S deficiency has been reported to protect the pancreas β cells from apoptosis and delay the development of streptozotocin (STZ)-induced type 1 diabetes mellitus [37]. HG is often used to induce type 2 diabetes; therefore, it can be seen from the above that the effect of H_2_S on different types of diabetes may be different.

Autophagy is also involved in diabetes. Liraglutide is an acylated glucagon-like peptide-1 analogue, which has a 97% amino acid homology with natural glucagon-like peptide-1 and has been widely used in the treatment of type 2 diabetes mellitus [38]. Liraglutide promotes autophagy and induces pancreatic β cell proliferation to improve diabetes in high-fat-fed or STZ-treated rats [39]. Recent studies indicate that H_2_S plays an important role by regulating autophagy in many diseases including cancer, tissue fibrosis diseases and glycometabolic diseases [9]; however, the relevant mechanisms have not been fully studied. In this review, we summarize the recent research on the role of H_2_S regulating autophagy in diabetic-related diseases to provide reference for future related research.

## 2. Exogenous H_2_S Plays an Important Role by Regulating Autophagy in Diabetic Cardiomyopathy

Diabetes affects the heart through a variety of mechanisms including metabolic disorders, abnormal subcellular components, microvascular damage and dysfunction of cardiac autonomic nerve damage [40,41,42]. Eventually, the structure and function of the heart are impaired, which is known as diabetic cardiomyopathy (DCM) [43,44,45,46]. DCM is characterized by myocardial fibrosis and myocardial cell loss and ventricular systolic and/or diastolic dysfunction, without coronary artery disease and hypertension [47,48]. Type 2 diabetes is characterized by protein misfolding and aggregation, leading to mitochondrial damage, excessive ROS production, apoptosis and ubiquitin aggregation [49,50]. ROS are a natural by-product of the normal metabolism of oxygen in healthy cells. Diabetes destroys the balance between ROS production and clearance, resulting in excessive production of ROS to damage cells [51]. Ubiquitin aggregation, mainly cleared by autophagy, can result in apoptosis and excessive ROS production [52,53]. Therefore, it can be inferred that promoting autophagy can improve DCM by decreasing apoptosis and ROS production via eliminating ubiquitin aggregation. Jichao Wu et al. found that in a DCM rat model, exogenous H_2_S could improve DCM through ameliorating diastolic function and increasing H_2_S production. Similar results were obtained in vivo. Mechanism research showed that exogenous H_2_S inhibited oxidative stress by decreasing ROS production and upregulating the expression levels of mitochondrial catalase (Mito-CAT) and manganese-dependent superoxide dismutase (Mn-SOD). The results of the experiment of the antioxidation mechanism showed that exogenous H_2_S had no significant effect on Nrf2 nuclear translocation, meaning it could be excluded that the antioxidant effect of exogenous H_2_S was mediated by the keap-1/Nrf2 signaling pathway. Exogenous H_2_S also increased autophagy by upregulating the expression level of Beclin1, microtubule associated protein 1 light chain 3 II (LC3II) and autophagy associated protein 7 (Atg7), promoting the degradation of autophagosome content and decreasing the expression level of p62. In order to explore a new explanation for the H_2_S antioxidant effect, the Keap-1 effect on promoting ubiquitin aggregate clearance was studied. The results showed that exogenous H_2_S decreased the ubiquitylation levels of Keap-1, CAT and SOD, which might be the reason why exogenous H_2_S had the antioxidant effect, while Keap-1 siRNA inhibited the effects of exogenous H_2_S on autophagy in the cardiomyocytes of diabetic rats. 3-MA (an autophagy inhibitor) abolished the antioxidant effect of exogenous H_2_S, suggesting that exogenous H_2_S promoted ubiquitin aggregation clearance by upregulating autophagy through activating keap-1. 1,4-Dithiothreitol, a reducing agent of disulfides, reduced the expression level of keap-1 by promoting its ubiquitination level and counteracted the effects of exogenous H_2_S on keap-1, ubiquitin aggregate clearance and oxidative stress in HG-induced H9C2 cells. Moreover, exogenous H_2_S could promote the formation of disulfide between two keap-1 molecules, suggesting that exogenous H_2_S suppressed Keap-1 ubiquitylation through promoting its disulfide formation. From the above results, it can be deduced that exogenous H_2_S ameliorates DCM by promoting ubiquitin aggregation clearance through promoting autophagy via ubiquitylation of Keap-1, which provides a new mechanism for the antioxidative stress of H_2_S [54]. In the above DCM models, whether endogenous H_2_S improves DCM through autophagy is worth studying. Moreover, more research is needed on how exogenous H_2_S inhibits keap-1 ubiquitination to promote autophagy.

Adenosine 5′-monophosphate activated protein kinase (AMPK) is a serine/threonine kinase and regulates many physiological and pathological processes including apoptosis, proliferation, cell growth, migration and differentiation [55,56]. The mammalian target of rapamycin (mTOR) is a serine/threonine protein kinase of the PI3K-related family, which regulates cell growth and metabolism in response to hormones and nutrition [57]. Our previous studies have shown that exogenous H_2_S inhibited NLRP3 inflammasome-mediated inflammation by activating autophagy via the AMPK/mTOR signaling pathway [58]. Similarly, exogenous H_2_S also can improve DCM by regulating autophagy through the AMPK/mTOR signaling pathway. Studies of Fan Yang and colleagues have shown that exogenous H_2_S could ameliorate left ventricular systolic dysfunction, increase the cell survival rate and inhibit cardiomyocyte apoptosis and oxidative stress to improve DCM. Meanwhile, exogenous H_2_S promoted autophagy by increasing autophagic vesicles and the expression level of autophagic-related proteins. Mechanism studies showed that exogenous H_2_S activated the AMPK/mTOR signaling pathway through increasing the p-AMPK/AMPK ratio and decreasing the p-mTOR/mTOR ratio in a diabetes model. Compound C, an inhibitor of AMPK, suppressed the activity of mTOR, suggesting that mTOR is downstream of AMPK in the regulation of autophagy by exogenous H_2_S. Compound C and AMPK-siRNA both inhibited H_2_S-promoted autophagy in HG-induced H9C2 cells, increased apoptosis and aggravated cell injury, indicating that exogenous H_2_S alleviated DCM by activating autophagy through activating the AMPK/mTOR signaling pathway [59]. The relationship between H_2_S-induced autophagy and the AMPK/mTOR signaling pathway needs further study in diabetes.

Myocardial fibrosis is caused by the excessive deposition of collagen in the extracellular matrix (ECM). It is one of the main pathological features of DCM, which can lead to diastolic and systolic dysfunction in patients with DCM [60,61]. Diabetes could promote myocardial fibrosis through increasing the cross-sectional area of myocardial cells, inducing the disorder of myocardial cell arrangement and promoting collagen deposition. Diabetes also increased the levels of hydroxyproline and the expression levels of collagen Ι, collagen III, transforming growth factor β1(TGFβ1) and matrix metalloproteinase (MMP) and decreased the expression level of tissue inhibitor of matrix metalloproteinase (TIMP) in the diabetic myocardium, while exogenous H_2_S reversed the diabetes-induced changes, suggesting that exogenous H_2_S could improve myocardial fibrosis induced by diabetes. Mechanism studies revealed that exogenous H_2_S inhibited diabetes-induced autophagy through decreasing the number of autophagosomes and the expression levels of Beclin-1, Atg3, Atg5 and Atg16. The expression of PI3K and AKT1 was inhibited by diabetes, while exogenous H_2_S reversed the changes, suggesting exogenous H_2_S activated the PI3K/AKT1 signaling pathway suppressed by diabetes [62]. It has been reported that the activation of the PI3K/AKT1 signaling pathway can inhibit autophagy [63]. Therefore, it can be deduced from the above that exogenous H_2_S mitigates diabetes-induced myocardial fibrosis by inhibiting autophagy via activating the PI3K/AKT1 signaling pathway, which needs to be further studied by using inhibitors to suppress autophagy and the signaling pathway.

Exogenous H_2_S can protect the myocardium by inhibiting overactivated autophagy in the HG environment. It has been reported that myocardial cell aging is closely related to myocardial fibrosis [64,65]. Yaling Li et al. found that HG promoted myocardial cell senescence by increasing the number of aged cardiomyocytes and the expression level of aging-related protein P16 in HG-induced H9c2 cells. HG also aggravated diabetic myocardial fibrosis by increasing the expression levels of type III collagen, MMP-8, MMP-13 and MMP-14 in the diabetic myocardium and inhibited autophagy by downregulating the expression levels of autophagy-related proteins including Atg5, Atg16L1 and Beclin1 in HG-induced H9C2. Meanwhile, exogenous H_2_S could reverse the above changes, indicating that exogenous H_2_S could improve diabetic myocardial fibrosis, inhibit myocardial cell senescence and promote autophagy in diabetes models [66]. In addition, it has been reported that the upregulation of autophagy inhibited myocardial cell senescence [67]. From the above, it can be deduced that exogenous H_2_S alleviates diabetic myocardial fibrosis through suppressing myocardial cell senescence via activating autophagy. Sirtuin6 (SIRT6), a member of the NAD^+^-dependent deacetylase family, has been reported to play an important role in senility [68]. AMPK and the AMPK-autophagy pathway were involved in senility [69]. Mechanism research showed that exogenous H_2_S activated the SIRT6/AMPK signaling pathway suppressed by HG through upregulating SIRT6 and AMPK protein expression, while the SIRT6 inhibitor or AMPK inhibitor reversed the effects of exogenous H_2_S on autophagy and senescence, suggesting that exogenous H_2_S could improve diabetic fibrosis by suppressing myocardial cell senescence via activating autophagy through activating the SIRT6/AMPK signaling pathway [66]. The reference needs to be studied by using an autophagy inhibitor to confirm the effects of autophagy on diabetic myocardial fibrosis.

## 3. Exogenous H_2_S Plays an Important Role by Regulating Autophagy in Diabetic Vascular Endothelial Cell Dysfunction

Endothelial cell dysfunction (ECD) can induce the impairment of endothelial cell barrier function and vasodilation and plays a vital role in the complications of diabetes [70,71]. It has been reported that the excessive production of ROS contributes to ECD by overactivating autophagy in diabetes [72,73,74]. The results of Jiaqi Liu and colleagues showed that exogenous H_2_S mitigated dysfunction of arterial endothelial cells by decreasing the expression levels of marker proteins including Von Willebrand factor (vWF), Integrin beta-1 (ITGβ1) and GP1β A and reduced apoptosis of rat aortic endothelial cells (RAECs) by decreasing the expression levels of apoptosis-related proteins and mitigating mitochondrial damage in a diabetes model. Meanwhile, N-acetylcysteine (NAC), a scavenger of ROS, had similar effects to exogenous H_2_S, indicating that ROS mediated the above effects of exogenous H_2_S. The LC3II/I ratio is an indicator of autophagy formation and maturity; Atg7 mediates the formation of LC3-II; Lamp2 is a lysosomal membrane protein, which can be used to monitor the fusion of autophagosomes and lysosomes. HG could increase the LC3II/I ratio and the expression levels of Atg7 and Lamp2 and decrease the expression level of P62 in HG-induced RAECs, while exogenous H_2_S reversed the changes. Moreover, the inhibition of autophagy with 3-MA and Atg7 siRNA reduced RAEC apoptosis induced by HG. From the above, it can be inferred that exogenous H_2_S improve HG-induced ECD through inhibiting autophagy induced by HG via reducing ROS production. Mechanism research showed that HG increased the ratio of p-AMPK/AMPK, while exogenous H_2_S reversed the change. Compound C also suppressed HG-induced autophagy, suggesting that exogenous H_2_S inhibited autophagy by suppressing the HG-induced AMPK signaling pathway [75]. One study indicated that the reduced ATP production activates AMPK, which promotes autophagy [76], while exogenous H_2_S ameliorated mitochondrial damage treated with HG by increasing ATP production, respiratory complex activity and the expressions and activities of SOD and CAT, suggesting that exogenous H_2_S inhibited HG-induced AMPK activation through improving mitochondrial damage [75]. Nrf2 is a key transcriptional regulator of antioxidant enzyme genes and is inhibited from transferring to the nucleus by coupling with Keap-1 in the cytoplasm [77,78]. The transfer of Nrf2 was not influenced by HG, while exogenous H_2_S promoted Nrf2 nuclear transfer [75]. In conclusion, it can be deduced that exogenous H_2_S improve ECD through inhibiting autophagy via the Nrf2-ROS-AMPK signaling pathway, which provides a new way to treat diabetes-induced ECD.

## 4. Exogenous H_2_S Plays an Important Role by Regulating Autophagy in Diabetic Renal Fibrosis

Diabetic nephropathy (DN) is the main cause of end-stage renal disease [79,80]. It is characterized by the accumulation of the extracellular matrix (ECM), which leads to progressive renal fibrosis, decreased renal function and irreversible tissue loss [81,82]. Lin Li et al. found that exogenous H_2_S ameliorated renal fibrosis by decreasing the expression levels of MMPs, collagen IV and TIMP in diabetic kidney tissue. Exogenous H_2_S also promoted autophagy by upregulating the expression levels of autophagy biomarkers, including LC3, Atg3, Atg5, Atg7, Atg12 and Atg16 [83]. Deregulated autophagy has been reported to be involved in renal fibrosis [84]. Therefore, it can be inferred that autophagy activation may mediate the effects of exogenous H_2_S on diabetes-induced renal fibrosis. Different from the above, another study showed downregulation of autophagy in diabetes-induced renal fibrosis [85]. This difference may be related to the course and stage of diabetic nephropathy. Moreover, exogenous H_2_S reduced the expression levels of TGFβ1, nuclear factor of kappa B (NF-κB) and AKT in diabetic kidney tissue, suggesting the TGFβ1, NF-κB and AKT pathways may mediate the effects of exogenous H_2_S on autophagy in improving diabetes-induced renal fibrosis [84], which needs further studies, such as studies using an inhibitor to suppress the TGFβ1, NF-κB and AKT pathways.

## 5. Exogenous H_2_S Plays an Important Role by Regulating Autophagy in Diabetic Macroangiopathy

Diabetic macroangiopathy can lead to cerebrovascular disease, which is one of the main causes of death in diabetic patients. Dysfunctional vascular smooth muscle (VSM) plays an important role in diabetic macroangiopathy [86,87]. The α-lipoic acid (ALA), a natural antioxidant synthesized by animals and plants, is a catalyst for oxidative decarboxylation of pyruvic acid and α- ketoglutarate [88]. Studies have shown that ALA inhibits the proliferation of vascular smooth muscle cells (VSMCs) and induces VSMC apoptosis through several signaling pathways [89,90,91]. Xuan Qiu and colleagues found that the levels of plasma H_2_S in diabetic patients and diabetic rats were decreased. ALA treatment could increase the level of plasma H_2_S in diabetic rats. ALA could also reverse the inhibitory effect of propargylglycine (PPG, an irreversible CSE inhibitor) on endogenous H_2_S production; furthermore, the expression level of CSE was decreased in diabetic rats, indicating that ALA could promote endogenous H_2_S production. ALA could protect VSMCs, while PPG reversed the ALA protective effect, suggesting that ALA protected VSM in diabetic rats by promoting endogenous H_2_S production. The expression levels of LC3BII/LC3BI, Beclin-1 and phosphorylated AMPK were increased, and the expression levels of p62 and phosphorylated mTOR were decreased in diabetic rats, while ALA could reverse the changes. Moreover, PPG attenuates ALA-induced inhibition of autophagy and the AMPK/mTOR pathway. Therefore, it can be inferred that ALA suppresses autophagy by suppressing the AMPK/mTOR pathway of VSM in diabetic rats through increasing the endogenous H_2_S level. Similar to in vivo experiments, exogenous H_2_S and ALA significantly inhibited autophagy and increased cell viability in HG-induced VSMCs. Rapamycin (an autophagy activator) and AICAR (an AMPK activator) both abolished the effects of exogenous H_2_S and ALA on autophagy and cell viability. In addition, AICAR activated AMPK and decreased the level of phosphorylated mTOR protein, and compound C further enhanced the above effects of exogenous H_2_S and ALA. Collectively, it can be deduced that exogenous H_2_S and ALA improve diabetic dysfunctional VSM by inhibiting autophagy via the AMPK/mTOR pathway, which offers a new strategy for the treatment of diabetic macroangiopathy [92].

## 6. Exogenous H_2_S Plays an Important Role by Regulating Autophagy in Diabetic Depression

Many studies have reported a high prevalence of depression in diabetic patients [93,94], and that H_2_S has antidepressant effects in diabetic rats [95]. Brain-derived neurotrophic factor (BDNF) is a neurotrophic factor, mainly expressed in the hippocampus and cortex, regulating the central nervous system [96], and plays neuroprotective effects through its high-affinity Tyrosine Kinase B(TrkB) receptor [97]. Moreover, it has been reported that BDNF promotes neuron survival through improving autophagy [98]. To prove whether H_2_S can improve diabetic depression by regulating autophagy, Hai Yao Liu and colleagues committed to a series of experiments, and the results showed that exogenous H_2_S increased the expression levels of BDNF and p-TrkB proteins in the hippocampus of diabetic rats. K252a (an inhibitor of the BDNF-TrkB pathway) abolished the antidepressant effects of H_2_S. Furthermore, K252a inhibited exogenous H_2_S-promoted hippocampal autophagy in diabetic rats by decreasing the number of autolysosomes and Beclin-1 expression and increasing P62 expression in the hippocampus of diabetic rats. In conclusion, exogenous H_2_S can improve diabetic depression by promoting autophagy via the BDNF-TrkB pathway [99], which provides a potential therapeutic value for depression caused by diabetes. The above conclusion still needs to be further studied by using an autophagy inhibitor, and in-depth research is needed to determine whether exogenous H_2_S can prevent diabetes depression by improving damaged hippocampal neurons and reducing synaptic plasticity-related proteins.

## 7. Conclusions

In this review, we summarized the role of exogenous H_2_S in regulating autophagy in diabetic-related diseases as follows: (1) exogenous H_2_S ameliorates DCM by promoting ubiquitin aggregation clearance through promoting autophagy via ubiquitylation of Keap-1; (2) exogenous H_2_S alleviates DCM by activating autophagy through activating the AMPK/mTOR signaling pathway; (3) exogenous H_2_S mitigates diabetes-induced myocardial fibrosis by inhibiting autophagy via activating the PI3K/AKT1 signaling pathway; (4) exogenous H_2_S could improve diabetic-induced myocardial fibrosis by suppressing myocardial cell senescence via activating autophagy through activating the SIRT6/AMPK signaling pathway; (5) exogenous H_2_S can improve HG-induced ECD through inhibiting autophagy via the Nrf2/ROS/AMPK signaling pathway; (6) exogenous H_2_S improves diabetes-induced renal fibrosis by activating autophagy via inhibiting the TGFβ1/NF-κB/AKT pathways; (7) exogenous H_2_S improves diabetic dysfunctional VSM by inhibiting autophagy via suppressing the AMPK/mTOR pathway; (8) exogenous H_2_S can improve diabetic depression by promoting autophagy via activating the BDNF/TrkB pathway (Table 1). From the above, we can see that in the improvement of diabetes, exogenous H_2_S sometimes activates autophagy and sometimes inhibits autophagy. The reason may be related to the severity of diabetes. In the early stage of diabetes, when the disease is mild, H_2_S often activates autophagy to protect cells, and with the progress of the disease, H_2_S inhibits the overactivated autophagy to protect cells. In addition, under physiological conditions, the level of autophagy in different tissues is different, which may be another reason for the abovementioned findings. The effect of autophagy on cells is a “double-edged sword” because if autophagy is maintained at a high level, autophagy will lead to autophagy death. Therefore, the role of autophagy in different complications of diabetes needs further study.

Our previous studies have shown that exogenous H_2_S can inhibit the inflammation of liver cells mediated by the NLRP3 inflammasome by upregulating autophagy [58]. Moreover, the NLRP3 inflammasome can regulate autophagy to play a role in diabetes [100]. Therefore, whether exogenous H_2_S can regulate autophagy through the NLRP3 inflammasome in diabetic-related diseases is a very worthy topic to study. With the further research on the improvement of diabetes by H_2_S regulating autophagy, it may provide a new strategy for the treatment of diabetes.

## Figures and Tables

**Table 1:** The mechanisms of the roles of exogenous H_2_S regulating autophagy in diabetic-related diseases.

The Name of Diabetic-Related Disease	Mechanism	Reference
Diabetic cardiomyopathy	Promoting ubiquitin aggregation clearance through promoting autophagy via ubiquitylation of Keap-1	[54]
Diabetic cardiomyopathy	Activating autophagy through activating AMPK/mTOR signaling pathway	[59]
Diabetes-induced myocardial fibrosis	Inhibiting autophagy via activating PI3K/AKT1 signaling pathway	[63]
Diabetes-induced myocardial fibrosis	Suppressing myocardial cell senescence via activating autophagy through activating SIRT6/AMPK signaling pathway	[66]
High glucose-induced endothelial cell dysfunction	Inhibiting autophagy via the Nrf2/ROS/AMPK signaling pathway	[75]
Diabetes-induced renal fibrosis	Activating autophagy via inhibiting TGFβ1/NF-κB/AKT pathways	[83]
Diabetic dysfunctional vascular smooth muscle	Inhibiting autophagy via suppressing the AMPK/mTOR pathway	[92]
Diabetic depression	Promoting autophagy via activating BDNF/TrkB pathway	[99]
